# Effect of introduction of indocyanine green angiography of parathyroid glands on postoperative hypoparathyroidism after total thyroidectomy

**DOI:** 10.1093/bjsopen/zrac059

**Published:** 2022-05-11

**Authors:** Marco Stefano Demarchi, Alexandros N. Flaris, Jordi Vidal Fortuny, Benoit Bedat, Wolfram Karenovics, Frederic Triponez

**Affiliations:** Thoracic and Endocrine Surgery and Faculty of Medicine, University Hospitals of Geneva, Geneva, Switzerland; Department of General, Digestive and Endocrine Surgery, Lyon Sud Hospital Center, Pierre Bénite, France; Department of Surgical Oncology, Lyon Sud University Hospital, Hospices Civils de Lyon, Lyon, France; Department of Surgery, Tulane University School of Medicine, New Orleans, Louisiana, USA; Thoracic and Endocrine Surgery and Faculty of Medicine, University Hospitals of Geneva, Geneva, Switzerland; Thoracic and Endocrine Surgery and Faculty of Medicine, University Hospitals of Geneva, Geneva, Switzerland; Thoracic and Endocrine Surgery and Faculty of Medicine, University Hospitals of Geneva, Geneva, Switzerland; Thoracic and Endocrine Surgery and Faculty of Medicine, University Hospitals of Geneva, Geneva, Switzerland

*Dear Editor*

Postoperative hypoparathyroidism is a frequent complication of total thyroidectomy occurring in as many as 30 per cent of thyroidectomy patients, with definitive hypoparathyroidism diagnosed in as many as 10 per cent^1,2^. Hypoparathyroidism can lead to cerebral, vascular, ocular, and renal damage, a significant reduction in quality of life, and a reduced life expectancy. To avoid iatrogenic hypoparathyroidism resulting from thyroidectomy, the anatomical position of the parathyroid glands and their vasculature must be fully understood.

Some centres including ours have explored the use of indocyanine green (ICG) dye for non-ionizing intraoperative parathyroid gland angiography^3–5^ Perfusion of the parathyroid gland correlates directly with function and preservation of one well perfused parathyroid gland (as indicated by ICG angiography) is sufficient to maintain a postoperative euparathyroid state^5^.

This retrospective observational single-centre study aimed to determine whether the introduction of ICG angiography resulted in a reduced incidence of hypoparathyroidism on postoperative day 1 (POD1).

Records of 1222 consecutive patients who underwent total thyroidectomy between January 2012 and November 2020 were reviewed. Patients with concomitant parathyroid disease as well as those undergoing completion thyroidectomies and/or revision of previous neck surgery were excluded. ICG angiography was performed as previously described, after the thyroid gland had been removed^5^.

The analyses included 428 patients who underwent total thyroidectomy before and 794 patients after the introduction of ICG angiography in September 2014. The two study groups did not differ significantly in terms of demographics (*Table S1*). POD1 hypoparathyroidism was diagnosed in 38 (9.3 per cent) patients before angiography and in 50 (6.4 per cent) patients after (difference 2.8 per cent, 95 per cent confidence interval (c.i.) −0.66 per cent to 6.3 per cent; *P* = 0.10, missing; *n* = 18 in both groups) (*Fig. 1*). The median serum parathormone (PTH) level was significantly higher in patients after the introduction of ICG angiography (median (interquartile range (i.q.r.)) 2.6 (1.80–3.60) pmol/l *versus* 3.17 (2.16–4.28) pmol/l; difference −0.56, 95 per cent c.i. −0.75 to −0.36; *P* < 0.001, missing; *n* = 18 before, *n* = 115 after). The mild reduction in calcium levels after the introduction of ICG angiography (corrected calcium median (i.q.r.) 2.27 (2.19–2.34) mmol/l *versus* 2.23 (2.16–2.30) mmol/l; difference 0.04 (95 per cent c.i. 0.02 to 0.06); *P* < 0.001, missing; *n* = 12 before, *n* = 113 after) is likely related to the fact that before ICG introduction the calcium supplementation after total thyroidectomy was systematic and this was not the case after 2014.

**Fig. 1:**
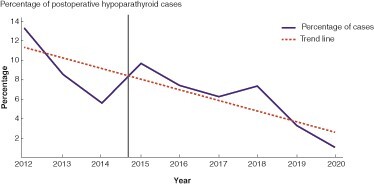
Line graph showing the percentage of patients with hypoparathyroidism (parathormone less than 1.1 pmol/l) for each year with the trend line (dashed line). Vertical line represents the date of introduction of indocyanine green angiography.

One possible explanation for the reduction in hypoparathyroidism reported in this study is that, in view of our previous results^5^, in some cases the thyroidectomy procedure was slightly modified. These modifications included dissections that were closer to the thyroid gland including some that were within the thyroid capsule. In some cases, a small remnant of thyroid tissue was left in place to preserve the vascular supply of the parathyroid glands. The progressive reduction of hypoparathyroidism could suggest that these modifications in the surgical technique occurred gradually, after the surgeons were confident with the results suggested by ICG angiography.

Another possible explanation is that ICG angiography might increase awareness of complications involving parathyroid gland preservation (the Hawthorne effect). Surgeons may have been alerted to the possibility of evaluating parathyroid glands with ICG angiography, and thus would subconsciously focus on a more meticulous dissection of the vascular pedicle.

The limitations of this study are mainly related to its retrospective nature. Because we compared data collected from two different periods, other factors may have contributed to the improved outcomes; however, the large number of patients and their comparable demographic characteristics, as well as the fact that the procedures were performed or supervised by only two senior surgeons who were already experienced at the beginning of the study interval, should reduce these biases.

## Funding

The study was funded entirely by the Department of Thoracic and Endocrine Surgery of Geneva University Hospitals.

## Acknowledgements

M.D. and F.T. were responsible for conceptualization. The methodology was developed by M.D. and F.T. All authors were responsible for data acquisition and validation. Formal analysis was conducted by M.D. and A.F. Investigations were conducted by M.D. Data curation was conducted by M.D. and A.F. M.D. drafted the original manuscript. M.D. and F.T. reviewed and edited the manuscript. All authors critically revised the manuscript, were responsible for visualization, supervision, and final approval, and read, and agreed to the published version of the manuscript.

*Disclosure*. F.T. received consulting fees from Stryker/Novadaq, Medtronic, and Fluoptics. The authors declare no other conflict of interest.

## Supplementary material

Supplementary material is available at *BJS Open* online.

## Data availability

Data are available on request from the corresponding author due to privacy restrictions. The data are not publicly available.

## Supplementary Material

zrac059_Supplementary_Data
