# Supplementary material for: Effect of introduction of indocyanine green angiography of parathyroid glands on postoperative hypoparathyroidism after total thyroidectomy
Source: BJS Open. 2022 May 11;6(3):zrac059. doi: 10.1093/bjsopen/zrac059 (PMC9092442; doi:10.1093/bjsopen/zrac059)
Supplement: zrac059_Supplementary_Data [file zrac059_supplementary_data.docx]

**Table S1.** **Characteristics of patients who underwent total thyroidectomy before and after the introduction of ICG angiography**

| **Variable** | **Before ICG introduction, N = 428** | **After ICG introduction, N = 794** | ***p*-value** |
| --- | --- | --- | --- |
| **Age, Median (IQR)** | 52 (22) | 51 (22) | 0.60 |
| **Sex, N (%)** |  |  | 0.40 |
| F | 352 (82%) | 637 (80%) |  |
| M | 76 (18%) | 157 (20%) |  |
| **Indication, N (%)** |  |  | <0.001 |
| Grave's disease/Toxic goiter | 112 (26%) | 220 (28%) |  |
| Nontoxic goiter | 162 (38%) | 290 (37%) |  |
| Retrosternal goiter | 5 (1.2%) | 44 (5.5%) |  |
| Suspicious nodule | 69 (16%) | 75 (9.4%) |  |
| Papillary thyroid carcinoma | 72 (17%) | 148 (19%) |  |
| Poorly differentiated thyroid cancer | 5 (1.2%) | 1 (0.1%) |  |
| Medullary thyroid carcinoma | 3 (0.7%) | 10 (1.3%) |  |
| Other | 0 (0%) | 6 (0.8%) |  |
| **Intervention, N (%) *(+(%)** |  |  | >0.99 |
| Total thyroidectomy | 358 (84%) | 665 (84%) |  |
| Total thyroidectomy with central neck dissection | 70 (16%) | 129 (16%) |  |

IQR: interquartile range
